# Localizing category-related information in speech with multi-scale analyses

**DOI:** 10.1371/journal.pone.0258178

**Published:** 2021-10-01

**Authors:** Sam Tilsen, Seung-Eun Kim, Claire Wang

**Affiliations:** Department of Linguistics, Cornell University, Ithaca, New York, United States of America; Universite Sorbonne Nouvelle Paris 3, FRANCE

## Abstract

Measurements of the physical outputs of speech—vocal tract geometry and acoustic energy—are high-dimensional, but linguistic theories posit a low-dimensional set of categories such as phonemes and phrase types. How can it be determined when and where in high-dimensional articulatory and acoustic signals there is information related to theoretical categories? For a variety of reasons, it is problematic to directly quantify mutual information between hypothesized categories and signals. To address this issue, a multi-scale analysis method is proposed for localizing category-related information in an ensemble of speech signals using machine learning algorithms. By analyzing how classification accuracy on unseen data varies as the temporal extent of training input is systematically restricted, inferences can be drawn regarding the temporal distribution of category-related information. The method can also be used to investigate redundancy between subsets of signal dimensions. Two types of theoretical categories are examined in this paper: phonemic/gestural categories and syntactic relative clause categories. Moreover, two different machine learning algorithms were examined: linear discriminant analysis and neural networks with long short-term memory units. Both algorithms detected category-related information earlier and later in signals than would be expected given standard theoretical assumptions about when linguistic categories should influence speech. The neural network algorithm was able to identify category-related information to a greater extent than the discriminant analyses.

## Introduction

What does it mean to “localize” category-related information in speech, and why is this a worthwhile goal? Theoretical models of speech often posit discrete categories, such as phones, articulatory gestures, syllables, moras, words, pitch accents, a hierarchy of phrase types, etc. Yet none of these theoretical entities is self-evidently “present” at any given point in time in acoustic or articulatory signals. How can we test whether speech signals contain evidence for these theoretical constructs, and if they do, how can we determine when in time that evidence is located? This is not a trivial problem and it requires some careful attention to our assumptions about the nature of speech and the concept of information.

One unavoidable issue in detecting evidence for theoretical categories in speech signals is that decisions must be made regarding the period of time in which to look for the relevant evidence. In this study, we propose a multi-scale analysis approach that does not require potentially arbitrary decisions regarding the period of time which is analyzed. Instead, analyses are conducted over a range of windows which are varied systematically in size and location. For each analysis window, a machine learning algorithm is trained to learn input-output mappings, and classification accuracy on unseen data is recorded. This training-testing procedure is repeated many times. In principle, any machine learning algorithm that is suitable for multidimensional continuous input variables and categorical outputs can be used. Here we examine two such algorithms: linear discriminant analysis (LDA) and deep neural network classification using two layers of bidirectional long short-term memory units (biLSTM).

To demonstrate the generality of the multi-scale analysis method, it is applied to two different datasets from separate experiments: a syllable production experiment and a relative clause production experiment. In both experiments, acoustic data were collected along with articulatory data, using electromagnetic articulography. In the syllable production experiment [1], a small set of consonant-vowel-consonant (CVC) syllables (e.g. *pop*, *pot*, *top*, *tot*, *op*, *…*) were repeatedly elicited from participants. The multi-scale analysis was used to infer when in these signals there is evidence for the onset consonant categories (p, t, and Ø = no onset). In the relative clause production experiment [2], two different types of relative clauses were elicited: restrictive relative clauses (RRC), e.g. *The Mr*. *Hobb* [*who knows Mr*. *Robb*] *often plays tennis*; and non-restrictive relative clauses (NRRC), e.g. *A Mr*. *Hobb*, [*who knows Mr*. *Robb*], *often plays tennis*. Multi-scale analyses were used to infer when in time there is evidence for the two relative clause types (RRC, NRRC).

The results show that category-related information is present in signals earlier and also later than is commonly assumed. In some cases, participants in the experiments exhibited distinct temporal distributions of category-related information. Furthermore, the neural network algorithm performed better than the linear discriminant analysis, particularly for shorter temporal windows. Below we elaborate on the notion of category-related information that is adopted in this paper and explain how the classification accuracy of a learning algorithm can be used in the localization task. We then describe the datasets and present the results and methods in detail. Finally, we discuss the implications of the findings and the potential uses of the multi-scale analysis method, along with its limitations.

### Category-related information

What do we mean by “category-related information” and how can such information be “localized”? Although not commonly described as such, there already exist speech analysis methods which in effect localize category-related information. A very common example is phone-based Hidden Markov Model (HMM) speech recognition [3–5]. The algorithms used in this context presuppose that speech is comprised of mutually exclusive, non-overlapping units called *phones*. These hypothesized units are hidden states of the model. The algorithms provide, for each frame of speech, the probability that the frame was produced (emitted) by each member of the set of possible phones (hidden states). The "frames" of speech in this case are small windows of the acoustic signal which have been transformed into feature vectors which encode spectral information. The focus of such algorithms is usually on the most probable segment, but the time-varying probabilities of the phone categories could be used to draw inferences regarding where in time those categories have a substantial influence on (i.e. “are present in”) the acoustic signal. For instance, in the word *teapot* during the vowel /i/ of *tea*, /i/ may be the most probable phone, but the upcoming phone /p/ may have some non-zero probability. Based on this hidden state probability, one could infer where in time (during which analysis frames) there is “evidence” for the /p/ category. Thus in speech recognition, HMM models implicitly perform a temporal localization, and this holds for more commonly used triphone-based models and models with additional levels of representation. However, HMM-based recognition models are probably not optimal for the localization task because of how they are trained: the information used for training is derived from *a priori* assumptions about where category-related information might be located in time.

Here we conceptualize the localization task from an information-theoretic perspective, where joint observations of categories and acoustic/articulatory signals could be assessed with regard to the mutual information between the categories and signals. This approach ultimately cannot be applied to speech without making problematic assumptions, but it is nonetheless instructive to relate the information-based conceptualization to the analyses that we conduct. Assume that we are observing one category out of some finite set of mutually exclusive categories. The act of observing a category produces information [6]. For a finite set of categories Y = {y_1_, y_2_, …, y_N_}, the information produced is the Shannon entropy of the probability distribution of categories, i.e. H(*Y*) = −∑_*i*_ p(*y_i_*)log p (*y_i_*), where p(y_i_) is the probability of category y_*i*_. For example, if the category members are the onset segments /p/, /t/, and /k/ in productions of the syllables /pa/, /ta/, and /ka/, and the probabilities of observing these segments are $\mathrm{Pr}(/p/)=\mathrm{Pr}(/t/)=\mathrm{Pr}(/k/)=\frac{1}{3}$, then the entropy of the distribution is $-\frac{1}{3}\log\left(\frac{1}{3}\right)-\frac{1}{3}\log\left(\frac{1}{3}\right)-\frac{1}{3}\log\left(\frac{1}{3}\right)=1.58$ bits. In a phenomenological sense, entropy is a quantification of uncertainty. When we observe a specific instantiation of the category, uncertainty is decreased to 0. Hence 1.58 bits of information is produced by observing a category in the above example—information is always associated with the resolution of uncertainty.

Consider, however, that the above reasoning presupposes that a category can be “observed”, which seems to contradict our claim that such entities are not self-evidently present in speech signals. The notion that categories can be observed must be interpreted as a hypothesis of a theoretical model, and in general we would like to evaluate the usefulness of such hypotheses. We hold that the extent to which “observing” a category provides information regarding other observations (such as speech signals) can be used to assess those hypotheses; likewise, the extent to which speech signals provide information regarding hypothesized categories can be viewed as a test of the hypotheses.

**Fig 1:**
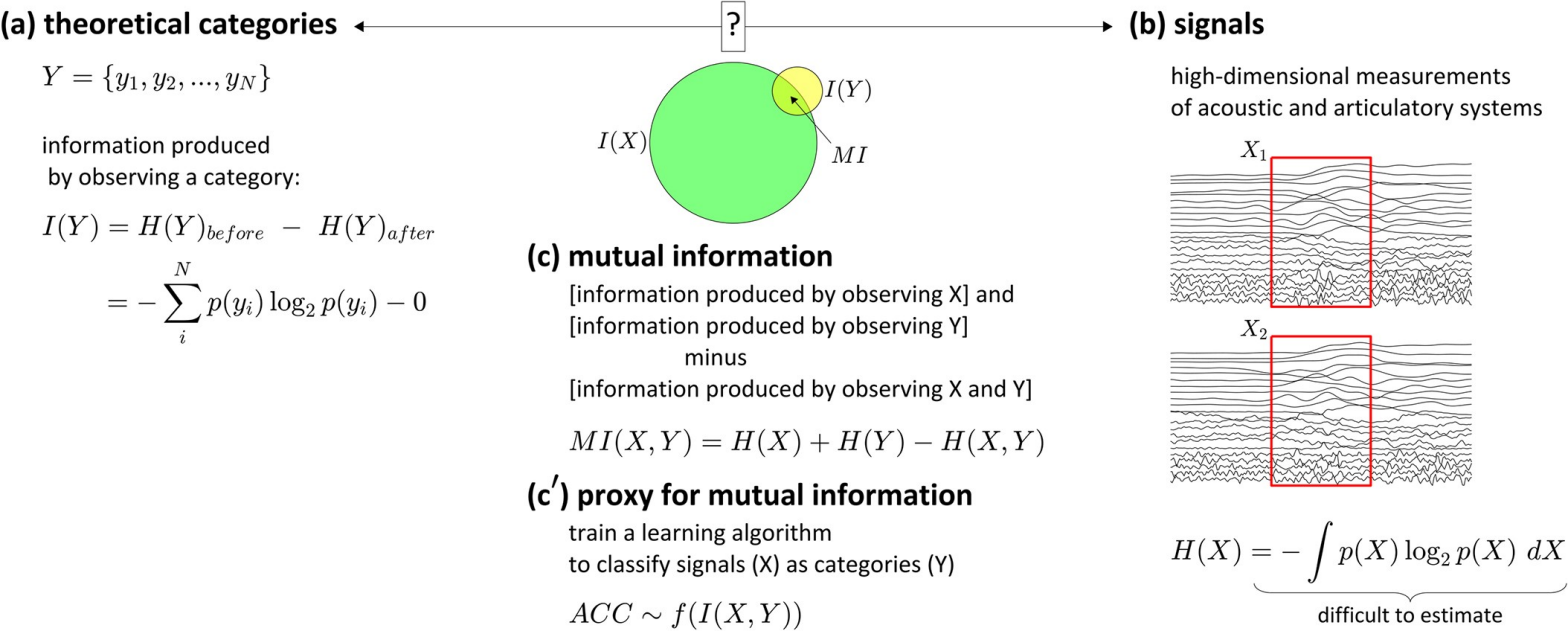
Information-theoretic interpretation of the task of localizing categories and signals. (a) Information produced by observing a category. (b) Information produced by observing signals. (c) Mutual information between categories and signals is calculated as the information produced by observing the signals and categories independently minus the information produced by observing them jointly. (c’) Because it is difficult to estimate the information of the signals, the classification accuracy of a learning algorithm with signals as input is treated as a proxy for mutual information.

The quantity that we would like to be able to calculate for these assessments—mutual information between hypothesized categories and signals—requires estimation of not only the entropy of the categories, but also the entropy of the signals and the joint entropy of the signals and categories. The mutual information between signals and categories is illustrated graphically in Fig 1 as the region of overlap between the information produced by observing the categories (Y) independently and the information produced by observing the signals (X) independently. The calculation of entropy for the theoretical categories is shown in Fig 1A, and the calculation of entropy for the signals is shown in Fig 1B, where probability functions are assumed to be high-dimensional and continuous.

However, estimating the entropy of multi-dimensional articulatory and acoustic speech signals over a period of time is problematic, for several reasons. First, the quality of the estimate depends on whether there is a sufficiently large ensemble of signals to accurately estimate probability distributions. Second, although there are many ways to obtain estimates of the relevant probability distributions [7, 8], these depend on smoothing or binning parameters. With high-dimensional signals many observations are needed for accurate estimation—unless bins are very large, much of the observation space will be unoccupied and estimates of the joint distribution will be unreliable. Third, the Shannon entropy does not take into account the dynamical evolution of processes, and thus is not wholly appropriate for time series in which current states depend on previous states. Schreiber [9] developed transfer entropy to incorporate transition probabilities from past states, and there are many extended conceptions of entropy for time-varying signals [10–12]. Transfer entropy is also equivalent to conditional mutual information computed on signals which have been subjected to time-delay embedding [13, 14]. However, in many cases such approaches assume the signals to be stationary (i.e. the statistical properties of the processes generating the signal do not change over time). In general, entropy estimation methods are most effective when time series are relatively long or when numerous observation sequences are available. Nonetheless, there are many novel approaches to analysis of time series which are designed to be suitable for nonstationary signals [12, 15, 16] or which do not require large datasets [14, 17, 18]. Although we do not apply such methods here, we suspect that it would be valuable to investigate their utility in relation to the goal of identifying speech categories.

Unlike other types of physiological signals (e.g. heart rhythms, gait, posture), speech signals collected in experiments are typically short and highly non-stationary. Thus it may not be appropriate to attempt to estimate the probability distributions that are required for calculating entropies. Moreover, signals observed from different speakers can have very different statistical properties, which entails that observations should not be combined across speakers, thereby resulting in practical limitations on the size of datasets that could be used for entropy estimation. For these reasons, the methods we examine here do not directly quantify information. However, it is worth pointing out there has been some success in applying methods that do in fact directly quantify information. One example is Iskarous et al. (2013) [19], where mutual information was used to measure coarticulation between adjacent segments. Such methods are most feasible when the probability estimates on which they are based are relatively accurate.

### Classification accuracy: An indirect solution

Instead of calculating information-theoretic measures, a more indirect but perhaps more robust approach is to use machine learning to infer the presence of category-related information. This inference is based on the ability of the learning algorithms to correctly classify categories in unseen data, after they have been trained on acoustic and articulatory signals which are hypothesized to be associated with those same categories. One way to motivate this machine learning approach is to assert that the "learning" that occurs is in and of itself a form of evidence that there exists some correspondence between observed signals and hypothesized categories. In other words, the fact that such algorithms can learn representations and transformations that allow for successful prediction with unobserved input signals and output categories constitutes evidence of correspondence between the signals and categories. Yet when reflecting on the meaning of "learning" and "representations" in this context, and on the conditions under which such learning is possible, we are drawn back to an information theoretic interpretation of learning in the classification task: the thing that is learned can be interpreted as a mapping between information in two domains, and the mapping that is learned is only generalizable when there exists mutual information between those domains. Of course, others researchers have drawn connections between machine learning and information theory (see e.g. [20]) and machine learning textbooks draw heavily on information-theoretic concepts, typically framing the learning process as one in which inputs are "information" [21]. We characterize our approach as indirect only because it does not involve an estimation of information-theoretic quantities, but at a fundamental level, the machine learning algorithms we are aware of can be interpreted from this perspective.

In our approach the presence of category-related information is assessed by whether algorithms learn generalizable mappings from high dimensional continuous input to low dimensional categories. Here we examine two different methods, linear discriminant analysis (LDA) and neural networks with bidirectional long short term memory units. It is not assumed that these are the optimal learning algorithms for current purposes, nor that the architecture and training parameters we use are optimal. To the contrary, we assume that these methods are sub-optimal and thus provide only lower bounds on the temporal extent of category-related information. Note that we use the phrase *category-related information* here because the relevant information that the algorithms use to classify inputs should be interpreted as information which is related to distinguishing theoretical categories. The method is agnostic regarding the causal mechanisms that are responsible for associations between hypothesized categories and signals, and there is no reason to assume that the relevant information is *specific* to the hypothesized categories.

The machine learning methods we employ to obtain classification accuracies can be given an information-based interpretation, even if the relation between classification accuracy and mutual information is ultimately indirect. In the case of LDA, the algorithm assumes each input dimension is Gaussian distributed and learns a function to divide the input space into regions associated with categories, such that the likelihood of the data given the categories is maximized. Consequently, the information produced by observing a category is minimized, if one already knows the data and the discriminant functions. In the case of the neural network algorithm, the information-based interpretation is less straightforward. It is not unusual to interpret the input-output mappings of neural networks from an information-theoretic perspective; indeed, it is common parlance to describe an “information flow” through the layers of a network (see for example [22]). Furthermore, it has been argued that classification networks *are* approximations to conditional distribution functions, albeit with a large number of parameters, and training such networks has been related to estimating maximal mutual information [23, 24]. Moreover, recent research has explicitly considered maximization of mutual information between input and output as a goal of network training [25, 26]. Thus the inferences we draw from network classification accuracy about category-related information are not wholly novel. It is also worth mentioning that our approach bears some similarities to the temporal generalization method developed in [27] and used recently in [28], although our approach differs by emphasizing analyses across a range of time scales and by restricting generalization to equivalent time epochs across signal observations.

The output of the method is a multi-scale analysis that we refer to as an *accuracy scalogram*, which is a representation of the average accuracy of a classification algorithm as a function of the center and size (i.e. scale) of the window of signal provided as input to the network. The term *scalogram* is borrowed from wavelet analyses, where wavelet transformations are applied to an input signal over a range of scales and locations. Some might consider this an abuse of terminology, but we suggest that the term is appropriate for this context as well. In all cases the analyses involve accuracy on unseen test data, thereby ensuring that the algorithms learn input-output mappings that are generalizable and not the result of overfitting.

**Fig 2:**
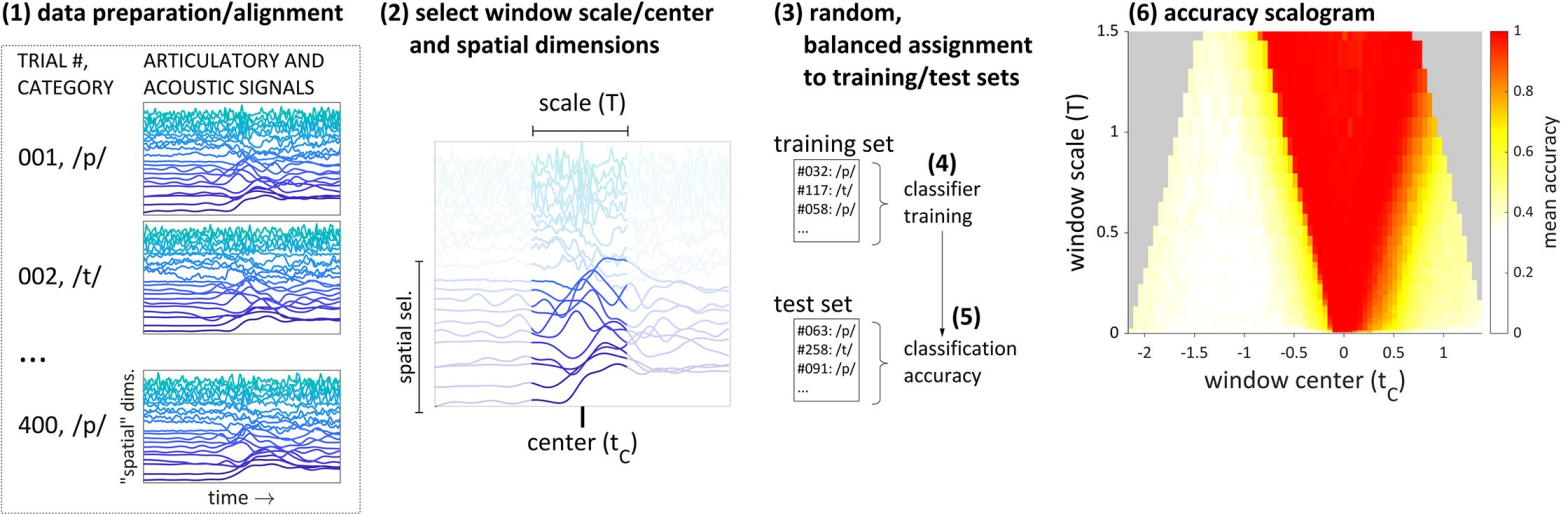
Overview of multi-scale analysis procedure. (1) observations of category-associated multi-dimensional time series are collected and aligned to a common event. (2) a window scale/center and spatial dimensions are selected. (3) observations are randomly assigned to training and test sets. (4) a discriminant classifier or neural network is trained to classify observations. (5) accuracy on the test set is recorded. Steps (3)-(5) are iterated for a range of window centers and scales, allowing for an accuracy scalogram to be constructed (6).

An overview of the procedure for generating accuracy scalograms is shown in Fig 2 with the following steps. Note that a more detailed description is provided in the *Methods* section. Step (1): an ensemble of multi-dimensional signals is collected, each of which is associated with a category (in this example phones, /p/ and /t/). The signals are aligned across observations to a common reference event that occurs in each observation. Step (2): the temporal and/or spatial dimensions of the input are restricted. The temporal restriction involves selecting a temporal window of the signal, which is defined by a center time *t*_c_ and a window size T (or, *scale*). The spatial restriction involves selecting a subspace of signal dimensions. Step (3): the observations are randomly partitioned into test and training subsets; categories are balanced in both sets. Step (4): a classifier is trained to classify the categories of the signals in the training subset. Step (5): after training, classification accuracy on the test subset (i.e. unseen data) is calculated. Steps (3)-(5) are repeated multiple times for a given analysis window, and the average classification accuracy on test data is calculated for that analysis window. Step (6): the procedure is repeated for a range of window centers and scales, and the mean accuracy for each window is presented in a scalogram.

### Interpreting scalograms

**Fig 3:**
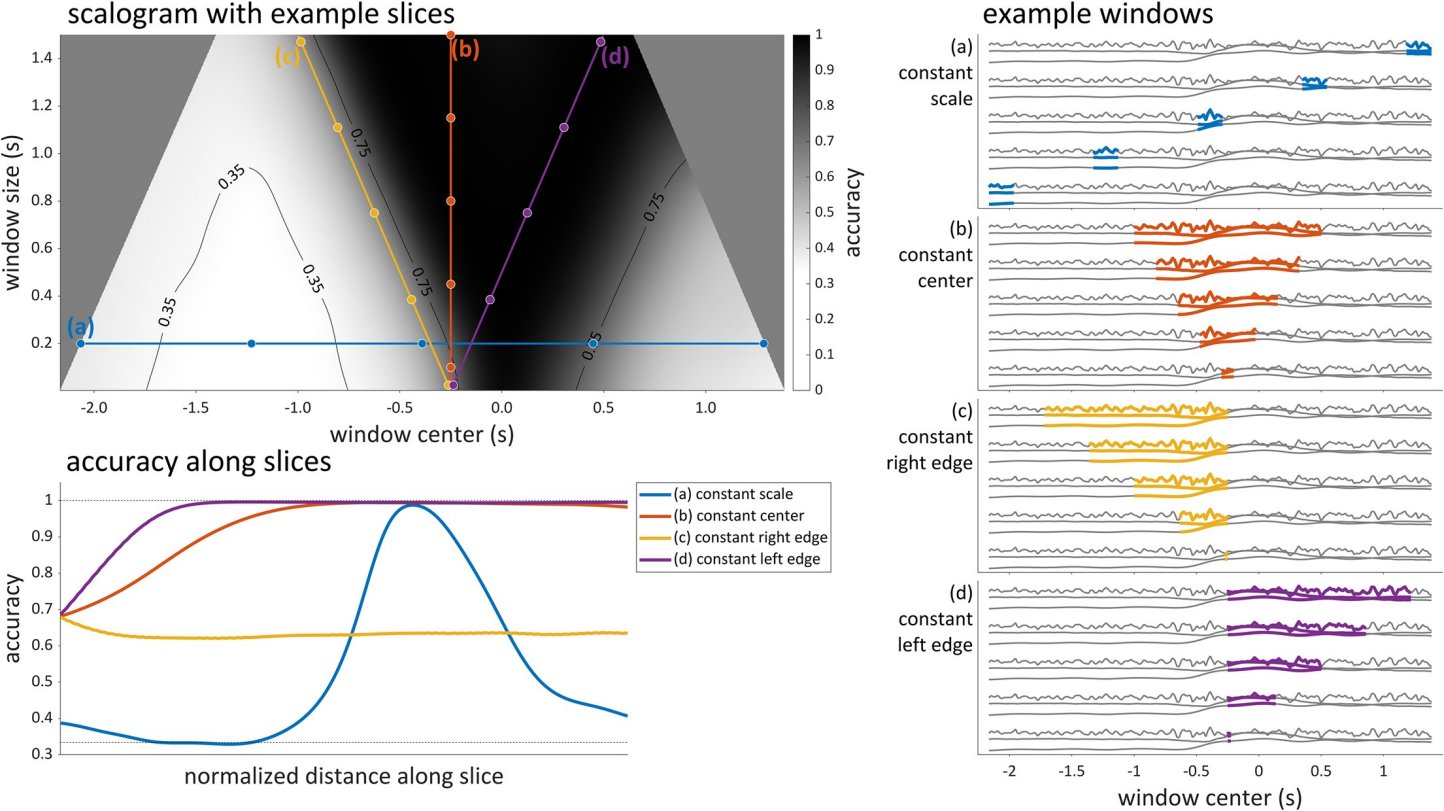
Example scalogram and interpretation. Top left: a smoothed, interpolated scalogram with four slices labelled (a)-(d), along with 35% and 75% accuracy contours. Bottom left: accuracies along each slice. Right panels (a)-(d): examples of signal windows for each of the five points in each slice.

Here we illustrate some aspects of scalograms that are relevant to their interpretation. The upper left panel of Fig 3 shows a smoothed and interpolated version of the scalogram in Fig 2. The shading of the scalogram represents the average classification accuracy on test data, for analyses conducted at each combination of window size (vertical dimension) and window center (horizontal dimension). In this particular example, we have used a grayscale colormap to represent accuracy, and chance performance is 1/3. The uniform gray triangular regions on the left and right of the scalogram represent analyses that are not conducted because they would require zero-padding of the input. The four lines labeled (a)-(d) in the scalogram represent slices of the scalogram in which some property of the analysis window is constant. For each slice, five points have been marked in the scalogram and the analysis windows corresponding to each of those points are indicated by the colored portions of the signals in the panels on the right. Specifically, in the constant scale slice (a), which corresponds to a horizontal line in the scalogram, window scale is the same for all analyses; hence each of the five windows shown in panel (a) is the same size (here 0.200 s). Note that in each of the panels (a)-(d) which show example windows, the same set of three-dimensional input signals is repeated five times, and the colored portions indicate the extent of signal that would be used for the particular window centers and scales indicated by the dots shown in the scalogram. The accuracy along slice (a) is shown in the lower left of Fig 3 (blue line). Notice that the accuracy along this slice is slightly above chance for analysis windows that occur early, falls to chance for windows that are centered a bit later, rises to near 100% accuracy around time 0.0, and then falls back down again. From this pattern we can infer that there is a lot of category-related information in the input around time 0.0, which is the alignment point in this example. We can also infer that there is some category-related information in the beginning of the signals.

Other slices in the figure illustrate alternative ways in which some aspect of the analysis window can be held constant. In the constant center slice (b), which corresponds to a vertical line in the scalogram, the center time of all windows is the same but their size is varied. By examining the accuracy along this slice, it is evident that as the window size increases, more category-related information is present. In the constant right edge slice (c), which corresponds to a line of negative slope in the scalogram, all analysis windows end at the same point in the time, but their centers and sizes vary. This is useful, for example, if one wants to know whether there is information regarding hypothesized categories that occurs before a particular time; in panel (c), observe that the example windows end at -0.250 s. If analyses conducted at any center/scale along this slice obtain above chance accuracy, we can infer that there is category related information prior to time -0.250 s. Similarly, slice (d) corresponds to a set of analysis windows which begin at the same time; this is useful for drawing inferences regarding the presence of category related information that is present after a particular time.

A general point to make regarding why the "multi-scale" nature of the scalogram is particularly appropriate for speech analysis derives from the fact that in many theoretical approaches, hypothesized linguistic units are hierarchically structured [29–32]. Because of this, categories have the potential to manifest across a range of temporal scales. For instance, it is a widely held belief that speech can be analyzed as a sequence of sounds (i.e. phonemes or segments) which are grouped into syllables, and in turn these are grouped into feet, which comprise words that are grouped into one or several phrasal levels, which in turn comprise utterances. Production models which attempt to describe mechanisms which operate across these scales [33, 34] at least in principle may allow for bidirectional interactions across scales. Scalographic analyses are well-suited to investigating such interactions because they do not require strong *a priori* assumptions about the relevant time scales over which the interactions might manifest.

There are a number of points that are useful to keep in mind when interpreting accuracy scalograms. A detailed discussion of these is provided in the section "General Discussion". Here we note the two most important ones. First, the machine learning algorithms we use cannot obtain reliably above-chance accuracy on test data when no category-related information exists in training inputs. In other words, the results are not due to overfitting. Second, the accuracies we obtain are necessarily lower bounds, because we cannot be sure that the analysis parameters are optimal. The space of parameters defining network architecture, training, and input signals is too large to conduct a systematic hyperparameter analysis; thus it is likely that for any given analysis, there is in fact a more optimal one which would obtain higher accuracy.

### Datasets and analyses

Two datasets are analyzed, one from a syllable production experiment [1], the other from a relative clause production experiment [2]. Limited analyses of category-related information in these datasets using neural networks have been reported in [1, 35]; here we present more comprehensive analyses which employ identical analysis parameters and more similar inputs, in order to facilitate more direct comparisons and to illustrate additional applications of the method. We also conducted linear discriminant analyses for comparison. In both datasets, acoustic and articulatory data were collected; articulatory data were obtained from electromagnetic articulography, which is an articulatory tracking method in which the positions of sensors adhered to articulators are recorded with high spatial and temporal resolution. Here the articulators included are upper lip (UL), lower lip (LL), jaw (JAW), tongue tip (TT), and tongue body (TB). The inputs to the learning algorithms are Mel-frequency cepstral coefficients (MFCCs) obtained from the acoustic signal, and articulator positions in the midsagittal plane; first differences of both signals are included as well. Further details regarding these inputs are in the *Methods* section.

For the syllable production dataset, the analyses focus on the identity of the pre-vocalic consonant, which belonged to one of three categories: {/p/, /t/, Ø}. The “Ø” refers to the absence of a phone in pre-vocalic position. The phone categories /p/ and /t/ are distinct with respect to the articulators that are used to form an oral closure: the lips for the /p/ and the tongue tip for /t/; the jaw is recruited for both sounds and the vocal folds are abducted when the sounds are in word-initial position. There were six participants in the experiment, all native speakers of English. On each experimental trial, production of a CVC syllable was cued by a visual signal, where C = {/p/, /t/, Ø} and V = /a/. Thus there were nine different targets, orthographically represented as: *pop*, *pot*, *pah*, *top*, *tot*, *tah*, *op*, *ot*, *ah*. Each target production was also preceded by the production of a prolonged /i/ vowel (for about 1.5 s), during which time the participant was informed of the identity of the target. It is important to keep in mind that prior to and during the pre-response vowel, participants were aware of the upcoming target. Participants performed 15 or 16 blocks of 36 trials, resulting in a total of 540 or 576 trials (50 or 54 trials per target form) per participant. The order of target forms was randomized within blocks. Further details regarding the experimental design can be found in [1].

The alignment point for the inputs in the syllable production analyses was the time of maximum tongue body speed associated with the tongue lowering/retraction from /i/ to /a/ postures. This event is a convenient alignment point because it is a relatively high velocity movement and is robustly detectable on all experimental trials. In the scalograms presented below, the alignment point is always defined as time 0. The leading edge of analysis windows which contain the point in time when the onset consonantal gesture has been initiated on 5% of trials is shown as well.

For the relative clause dataset, analyses focus on the identity of the relative clause category. Six native speakers of English participated in the experiment. The sessions alternated between blocks of trials in which one of two different types of relative clauses were elicited: a restrictive relative clause (RRC), e.g. *The Mr*. *Hobb* [*who knows Mr*. *Robb*] *often plays tennis*, or a non-restrictive relative clause (NRRC), e.g. *A Mr*. *Hobb*, [*who knows Mr*. *Robb*], *often plays tennis*. The sentences were always the same except for the pre-boundary names (*Hobb*, *Robb*, *Lobb*, *Hodd*, *Rodd*, *Lodd*) and the utterance final nouns, which were randomized from trial to trial. Cues were also presented to induce variation in speech rate in the experiment. The RRC vs. NRRC contrast may be associated with a difference in prosodic phrase structure that is manifested in pre- and post-clause boundaries. Specifically, it has been hypothesized that the NRRC is associated with a higher-level prosodic phrase than the RRC. Such phrasal differences are usually manifested in articulatory and acoustic changes in the temporal vicinity of phrase boundaries [36–39]. Further details regarding the experimental design are in [2].

The alignment points for inputs in the relative clause analyses were the pre-clausal and post-clausal boundaries, which were determined from forced alignment of the acoustic signal. Separate scalographic analyses were conducted for the pre- and post-clausal boundaries. Because speech rate was manipulated in the relative clause production experiment, the degree to which signals become misaligned with distance from the alignment point is greater than it is for the syllable production task. Hence the temporal range of analyses of the relative clause data is smaller.

For both datasets, all analyses are conducted within participant, in order to allow for better classifier performance. The motivation for conducting only within participant analyses is that there is substantial variation in vocal tract geometry between speakers, and this leads to variation in both articulatory and acoustic signals. This form of variation would likely have a negative impact on classification accuracy if the data were grouped across participants, although it remains to be determined how large this impact might be. Note that chance accuracy is 1/3 in the syllable production dataset because there are three categories (/p/, /t/, Ø), and 1/2 in the relative clause dataset because there are two categories (RRC, NRRC).

## Results and discussion

In the following presentation of results, we first compare the neural network and linear discriminant analyses on the syllable production dataset. Because the network-based classification algorithm outperformed the discriminant based one, subsequent analyses of temporal localization and signal dimension redundancy focus on the network-based results.

### Comparison of classification algorithms

**Fig 4:**
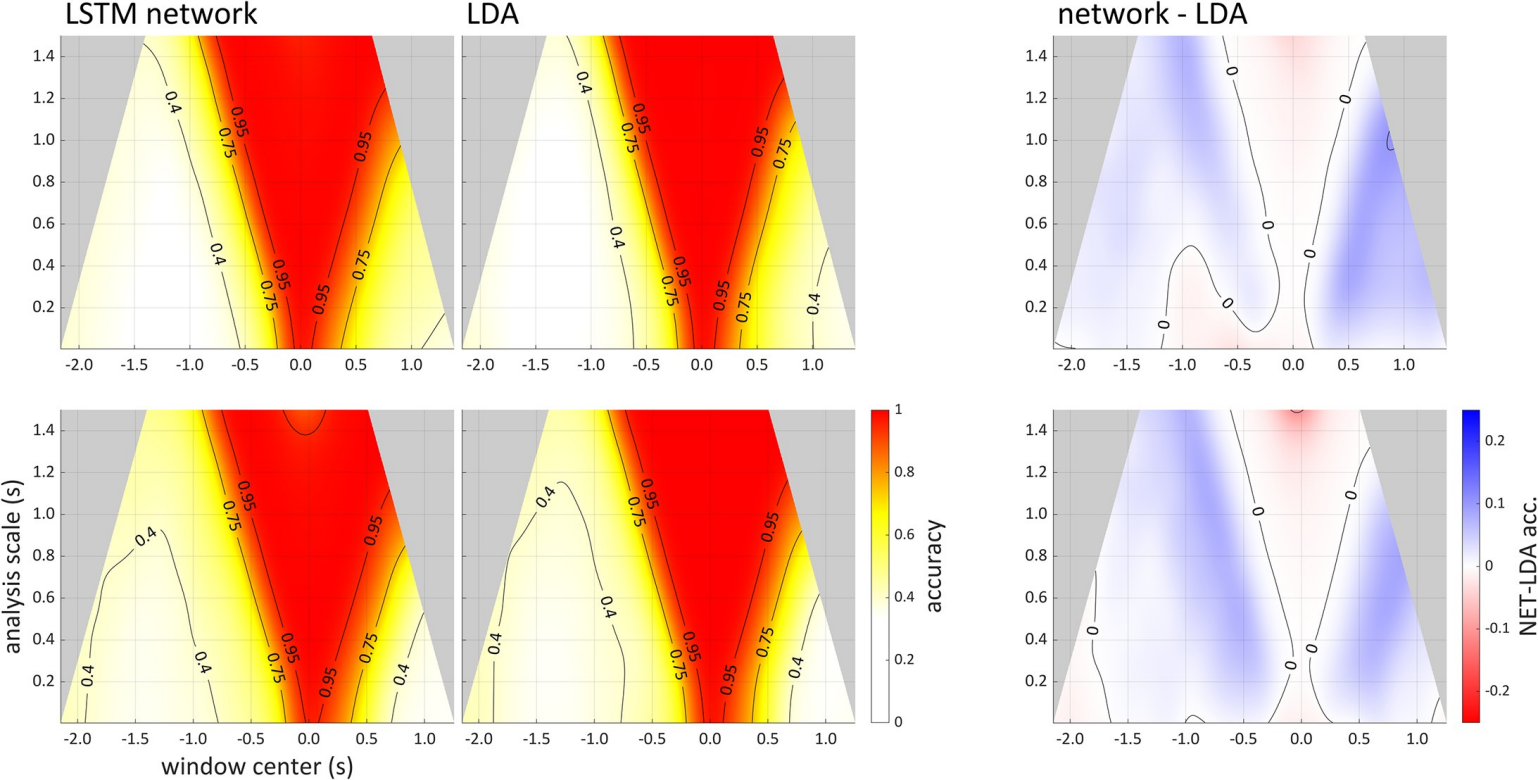
Scalographic comparison of neural network and LDA algorithms. Top and bottom panels are from participants sy01 and sy06 respectively in the syllable production dataset. Light gray areas are analyses which are not conducted because they would extend beyond the range of available signal. Left: scalograms of neural network and LDA analyses. Right: difference between neural network and LDA accuracies; where blue indicates that the neural networks achieved higher accuracy than LDA.

The neural network algorithm modestly outperformed linear discriminant analyses, but only for small to medium window sizes. At relatively large window sizes the neural network performance degraded. Fig 4 shows scalographic summaries of neural network and discriminant accuracies from two participants in the syllable production dataset, along with the difference between these accuracies. Here and elsewhere we present smoothed, interpolated scalograms obtained using procedures described in the Methods Section. Contour lines for 0.4, 0.75, and 0.95 accuracies are shown in the scalograms. As expected, in both analyses, the highest accuracy for a given scale (a horizontal slice) is achieved in the temporal vicinity of the onset of the vocalic movement (time 0), which is often about halfway between the formation and release of the consonantal closures for /p/ and /t/.

The panels on the right side of the figure show the difference between analyses (network—LDA), where blue corresponds to a positive difference, i.e. higher accuracy for the network analyses. At medium scales (up to about 1 s), it is evident that the network analysis achieved somewhat higher accuracy. For some windows, the differences approached 10%. However, at larger scales (near 1.5 s) network accuracy degrades substantially in comparison to the discriminant analysis. As we discuss later, this degradation is likely due to the network architecture and/or training procedures (section 3.4), and we note that for scales above 1.5 s (not shown in Fig 4) this degradation was quite substantial.

**Fig 5:**
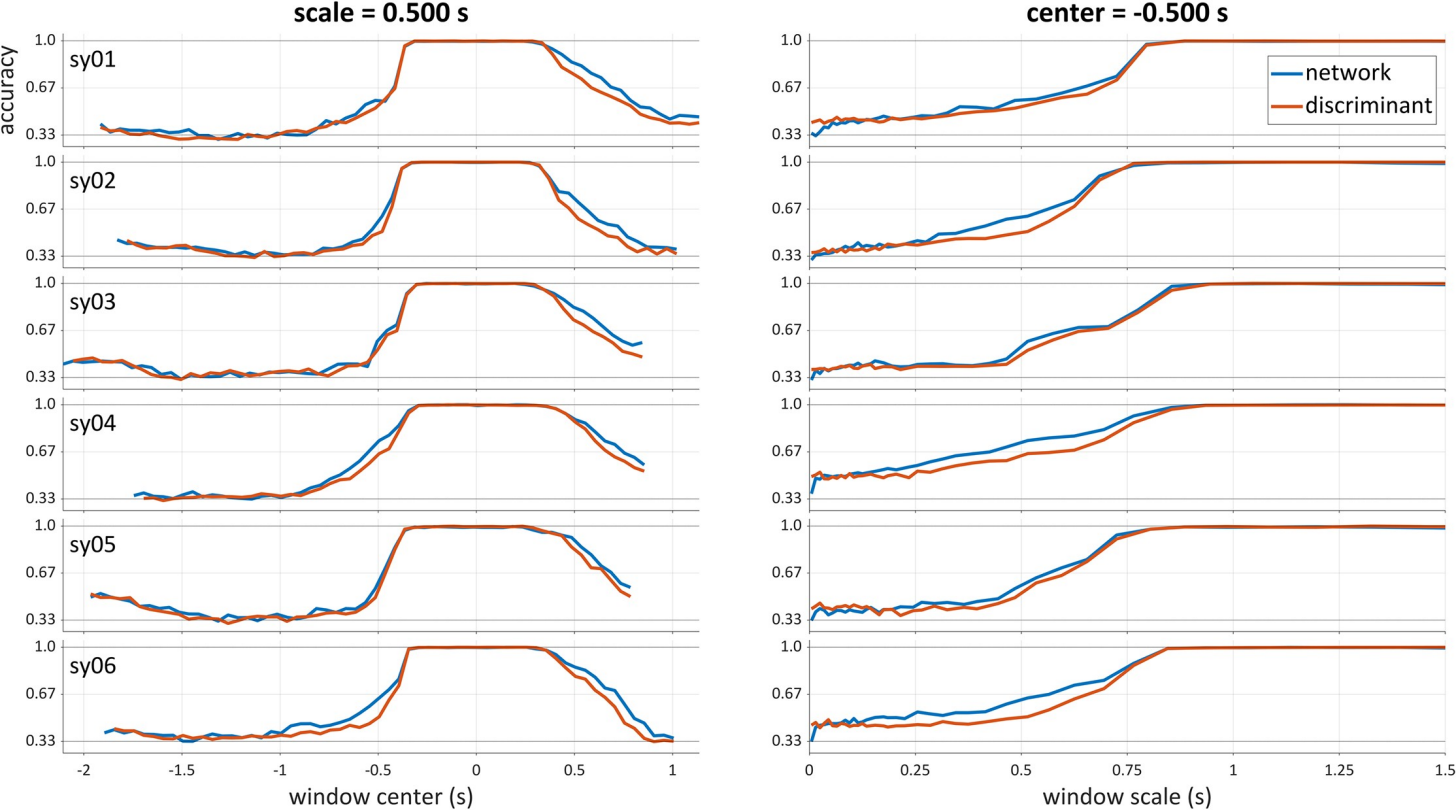
Comparison of accuracies of neural network and discriminant analyses for fixed scale and center. All six participants from the syllable production dataset are shown. Left: average accuracies as a function of window center, at scale 0.500 s. Right: accuracies as a function of window scale, at center -0.500 s. Blue line: network accuracy; orange line: linear discriminant accuracy.

To further illustrate differences between network and discriminant analyses, accuracy functions from both analyses are plotted for each participant in Fig 5. The panels on the left show accuracies as a function of window center for a fixed analysis scale of 0.500 s (i.e. a horizontal slice through the raw scalogram). The panels on the right show accuracies as a function of window scale for windows centered on time t = -0.500 (i.e. a vertical slice through the scalograms). Note that the accuracy functions shown here are not smoothed and interpolated.

For the constant-scale accuracy functions (Fig 5, left), it is clear that biggest advantages of the network analyses over LDA are present before and after the alignment point. For the constant center functions (Fig 5, right), the network outperforms LDA over the range of scales from 0.200–0.800 s.

### Temporal distribution of category-related information

**Fig 6:**
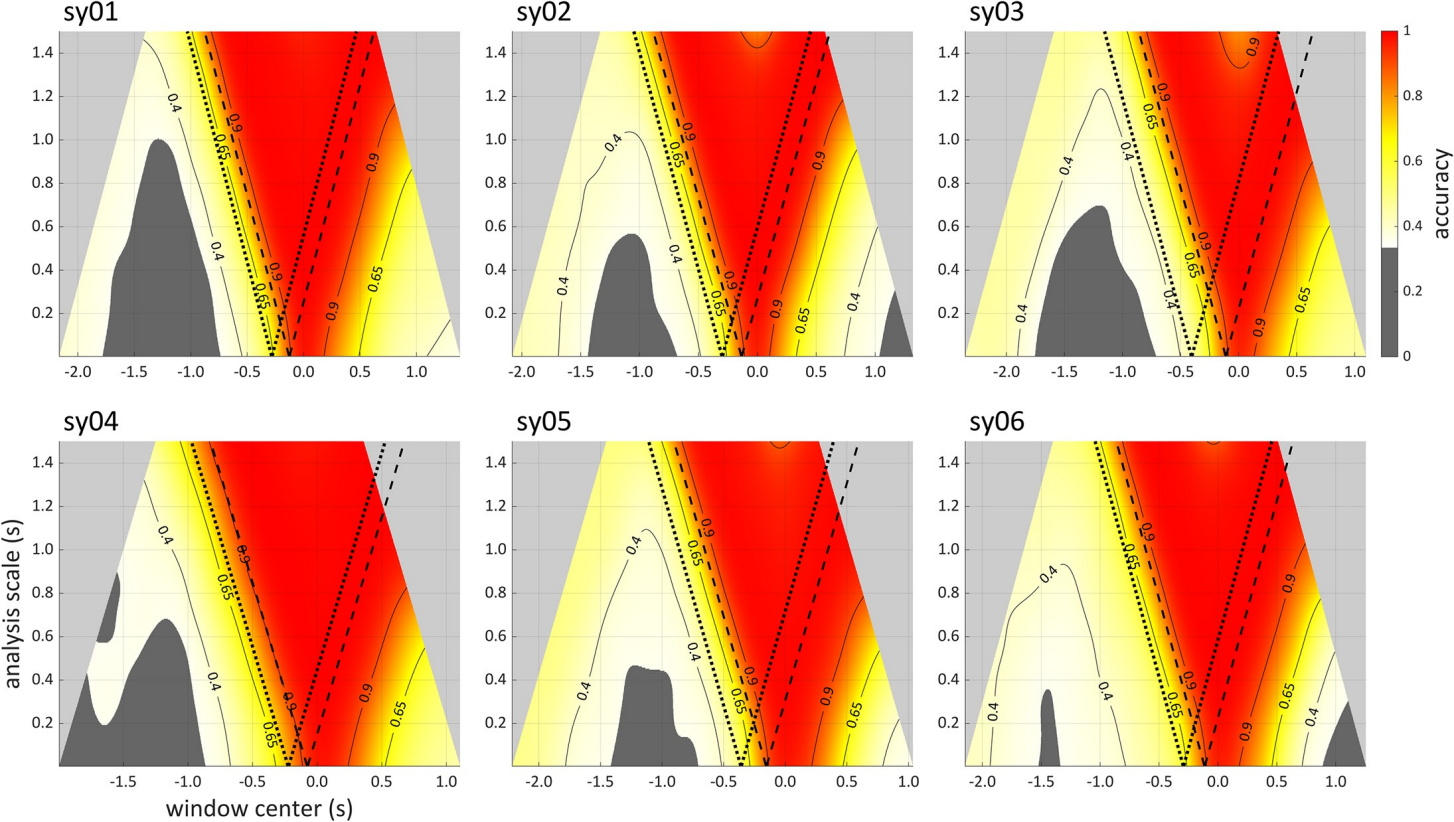
Accuracy scalograms for all six speakers in the syllable production dataset. Scalograms are smoothed and interpolated. Dark gray areas indicate where accuracy is not significantly above chance (1/3). The pairs of dotted and dashed lines in each panel show the centers of windows whose right or left edges align with the 5^th^ and 95^th^ percentiles of distributions of onset consonant movement initiation. The dotted lines on the left are associated with windows that end before the time of movement initiation, and the dotted lines on the right are associated with windows that begin at the time of movement initiation.

Multi-scale analyses are useful for drawing inferences regarding the temporal distribution of category-related information. Scalograms of neural network accuracy for all six participants in the syllable production dataset are shown in Fig 6. In each panel, the horizontal dimension is window center and the vertical dimension is window scale. The pairs of dotted and dashed lines in each panel indicate the centers of windows whose edges align with the 5^th^ and 95^th^ percentiles of distributions of movement initiation times associated with the onset consonants /p/ and /t/. The pair of dotted and dashed lines on the left of each panel is associated with centers of windows whose endpoints are located at the movement initiation percentiles, and the pair of dotted and dashed lines on the right is associated with windows whose start points are located at the 5^th^ and 95^th^ percentiles. In other words, the dotted lines on the left represent analyses of signals that precede movement initiation on 95% of trials, and the dotted lines on the right represent analyses which begin at movement initiation or earlier on 95% of trials (see Fig 3 for additional explanation of how to interpret these lines). Regions of the scalograms where the 95% confidence interval is below chance accuracy (< 1/3) are shaded dark gray.

**Fig 7:**
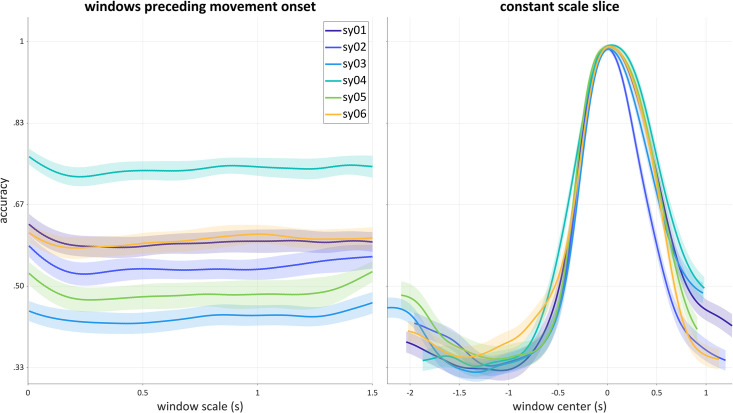
Accuracy slices for all six speakers in the syllable production dataset. Left: accuracy as a function of scale for windows whose right edge is aligned with the 5^th^ percentile of onset consonant movement initiation (dotted lines in Fig 6). Right: accuracy as a function of window center with constant scale of 0.250 seconds.

There are a number of interesting inferences that can be drawn from visual inspection of the scalograms. First, in all cases the network classifier can achieve substantially above-chance accuracy using signal windows that precede the 5^th^ percentile of the movement onsets. To reinforce this point, Fig 7 (left) shows accuracies as a function of scale, for windows whose right edge is located at the 5^th^ percentile of movement initiation for each speaker. In all cases these analyses exhibit above-chance accuracy. This is unexpected from conventional approaches to analysis of articulatory movements, such as the Task Dynamic model [1, 40], in which constriction movement onset should be the earliest observable correlate of an onset consonant. It is also unexpected in the DIVA model, in which acoustic effects of segments cannot occur before they exert influences on motor commands [41]. One plausible interpretation of this effect is that speakers are adjusting the posture of their vocal tract in a response-specific way, well before the canonical onset of movement. Of course, the existence of long-distance coarticulatory phenomena and phonological patterns [42–46] suggests that such effects should exist. Nonetheless, models of articulatory control have not yet attempted to account for them [34, 46].

Second, for most of the speakers, there is an unexpected region of above-chance accuracy obtained from analyses of early portions of the input. For instance, if one examines a horizontal slice at a scale of 0.250 s, accuracy is higher at the earliest windows (circa -2.0 s), decreases to chance levels between -1.5 to -1.0 s, and subsequently increases. To illustrate this more clearly, Fig 7 (right) shows accuracy as a function of window center, for analyses with a window size of 0.250 (i.e. a constant scale slice). This pattern suggests that the influence of the target onset consonant on the pre-response vowel is stronger earlier in the pre-response vowel. This is unexpected because the initiation of the response is about two seconds in the future for the earliest windows. This effect could be due to the fact that the target stimulus has become visible more recently during very early windows, or because the earlier portion of the pre-response vowel is more susceptible to an influence of the upcoming target.

Third, for all speakers substantially above-chance accuracy is obtained subsequent to the onset consonant, in most cases up to 1.0 s after the vocalic movement has been initiated. This indicates that the onset consonant in a CVC syllable can have effects on the coda consonant and beyond. It is worth noting that such effects—i.e. onset-coda interactions within a syllable—have rarely been investigated phonetically and are not well accommodated by current models (see [1] for further discussion of this point).

**Fig 8:**
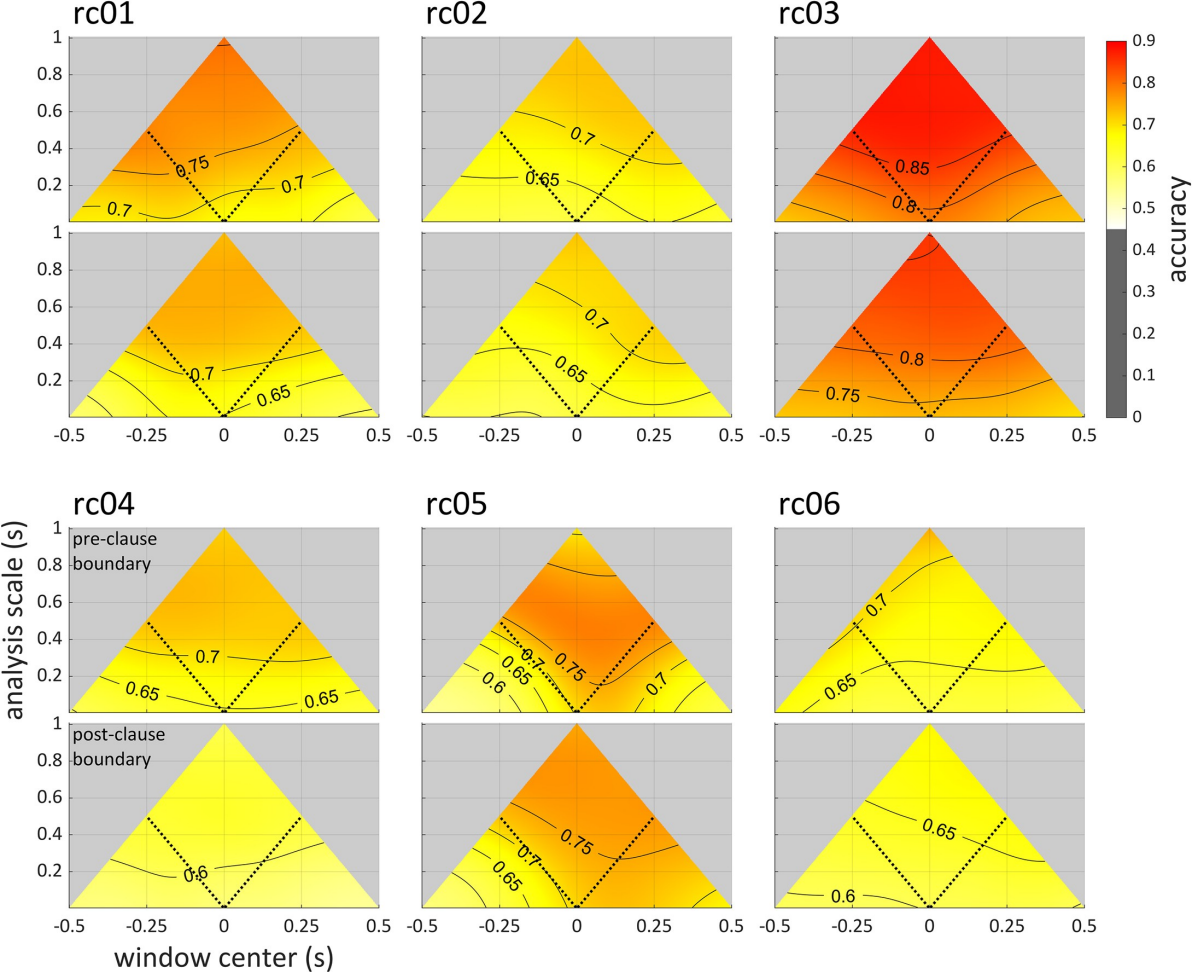
Accuracy scalograms for all speakers in the relative clause dataset. Scalograms are smoothed and interpolated. The alignment points (time 0) are the pre- and post-clause boundaries, for example: *A Mr*. *Hobb* |_pre_ *who know Mr*. *Rodd* |_post_ *often plays tennis*. The dotted lines in each panel show the centers of windows whose edges align with the boundaries.

Another way in which the distribution of information can be characterized from scalographic analysis relates to temporal asymmetries in accuracy. Scalograms of network accuracy at the pre-clause and post-clause boundaries for all participants in the relative clause dataset are shown in Fig 8. Significantly above-chance accuracy (> 1/2) was observed in all analysis windows within ±0.500 s of the boundary (time 0); however, near-maximum accuracy was never achieved. In comparison with the syllable production analyses, it is tempting to infer that there is “less information” regarding relative clause categories in the acoustic and articulatory signals than there was for the onset categories; however, this interpretation is not justified because there is no guarantee that the network algorithm performs equally well in both datasets. A more justifiable inference that can be drawn in this case involves temporal asymmetries in network accuracy within the relative clause dataset. For example, accuracies for rc02 and rc05 are generally higher in analysis windows that follow the pre- and post-clause boundaries than they in windows that precede those boundaries. In contrast, for rc01 accuracies are higher in windows that precede the pre- and post-clause boundaries.

**Fig 9:**
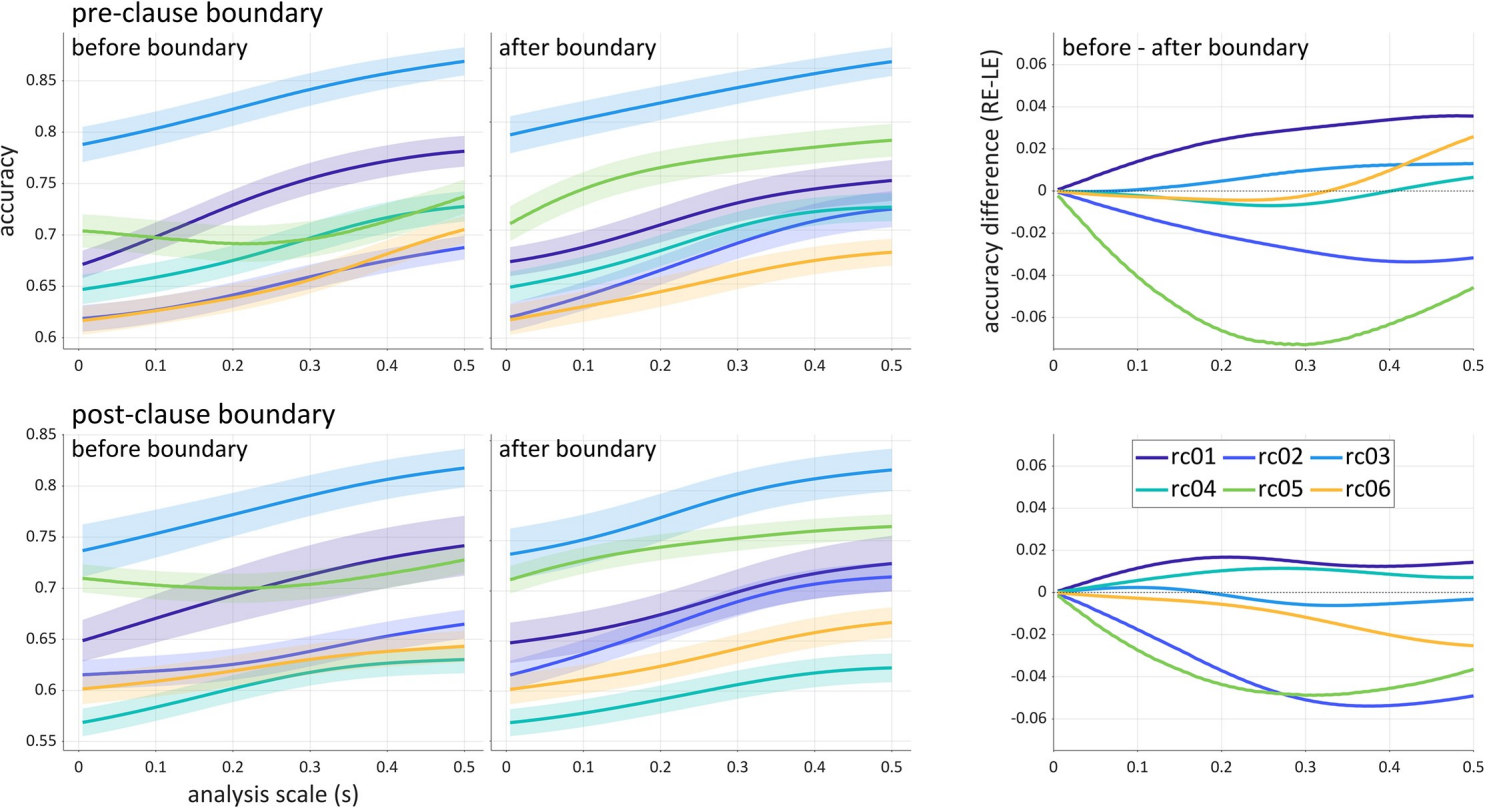
Comparison of accuracies before and after clause boundaries. Left panels: average accuracies as a function of scale for windows whose right or left edges (RE/LE) align with the pre- or post-clause phrase boundary. Filled regions are ±1.0 standard error. Right panels: difference in average accuracies (RE aligned windows–LE aligned windows). The alignment points (time 0) are the pre- and post-clause boundaries, for example: *A Mr*. *Hobb* |_pre_ *who know Mr*. *Rodd* |_post_ *often plays tennis*.

The temporal asymmetries in the distribution of relative clause related information are shown more directly in Fig 9, which plots the average accuracies of windows whose right and left edges align with the pre-clause boundary (top) and post-clause boundary (bottom). These are the analyses along the diagonal lines in Fig 8. The panels on the right of Fig 9 show the difference of the average accuracies. Note that at very small scales, the window centers are close in time and hence have very similar accuracies.

As is evident from Fig 9, for two speakers (rc02, rc05) higher accuracies are obtained in windows that follow both boundaries. This suggests that for these speakers, the phonetic manifestations of the syntactic contrast are expressed after boundaries to a greater extent than they are before the boundaries. For one speaker (rc01), pre-boundary windows provide higher accuracy. This suggests that for this speaker, more relevant information was present prior to the beginning of the relative clause than subsequent to the beginning of the clause, and likewise more information was present before the end of the clause than after the end of the clause. For rc06, differences become more evident as analysis scale increases and are dissociated between boundaries: more information precedes than follow the pre-clause boundary, but the reverse holds for the post-clause boundary. The interspeaker differences are of theoretical interest because they indicate that speakers may adopt different strategies for prosodically cueing syntactic contrasts.

### By-category accuracy analyses

**Fig 10:**
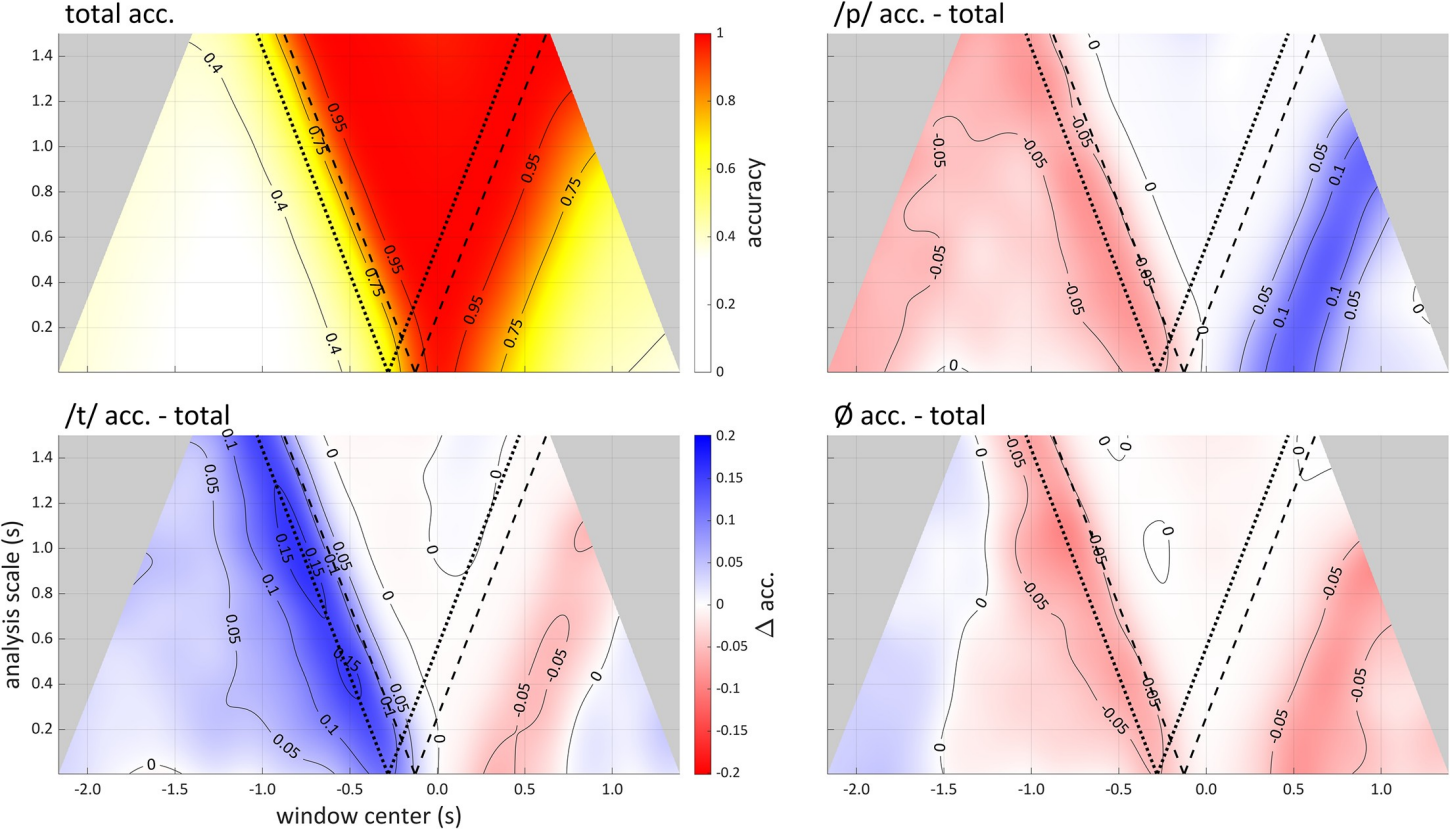
By-category analysis with accuracy scalograms from speaker sy01 of the syllable production dataset. Scalograms have been smoothed and interpolated. Upper left): mean accuracy. Other panels: difference between category-specific accuracy and total accuracy. Blue areas indicate higher within category accuracy than total accuracy.

Accuracy scalograms can be used to infer whether there are between-category differences in the temporal distribution of category-related information. Fig 10 shows differences in by-category accuracy and total accuracy for each of the three categories in the syllable production dataset. These comparisons suggest that /t/-related information is present before movement initiation to a greater extent than /p/- or /Ø/-related information. A possible explanation for this involves the fact that the primary articulators for /t/ and /p/ are the tongue tip and lips, and the primary articulator for the pre-response vowel is the tongue body (recall that speakers produce a prolonged /i/ before the target). The tongue tip interacts more strongly with the tongue body than the lips, due to their biomechanical coupling; hence more /t/-related information might be expected prior to the target response.

### Signal dimension redundancy

**Fig 11:**
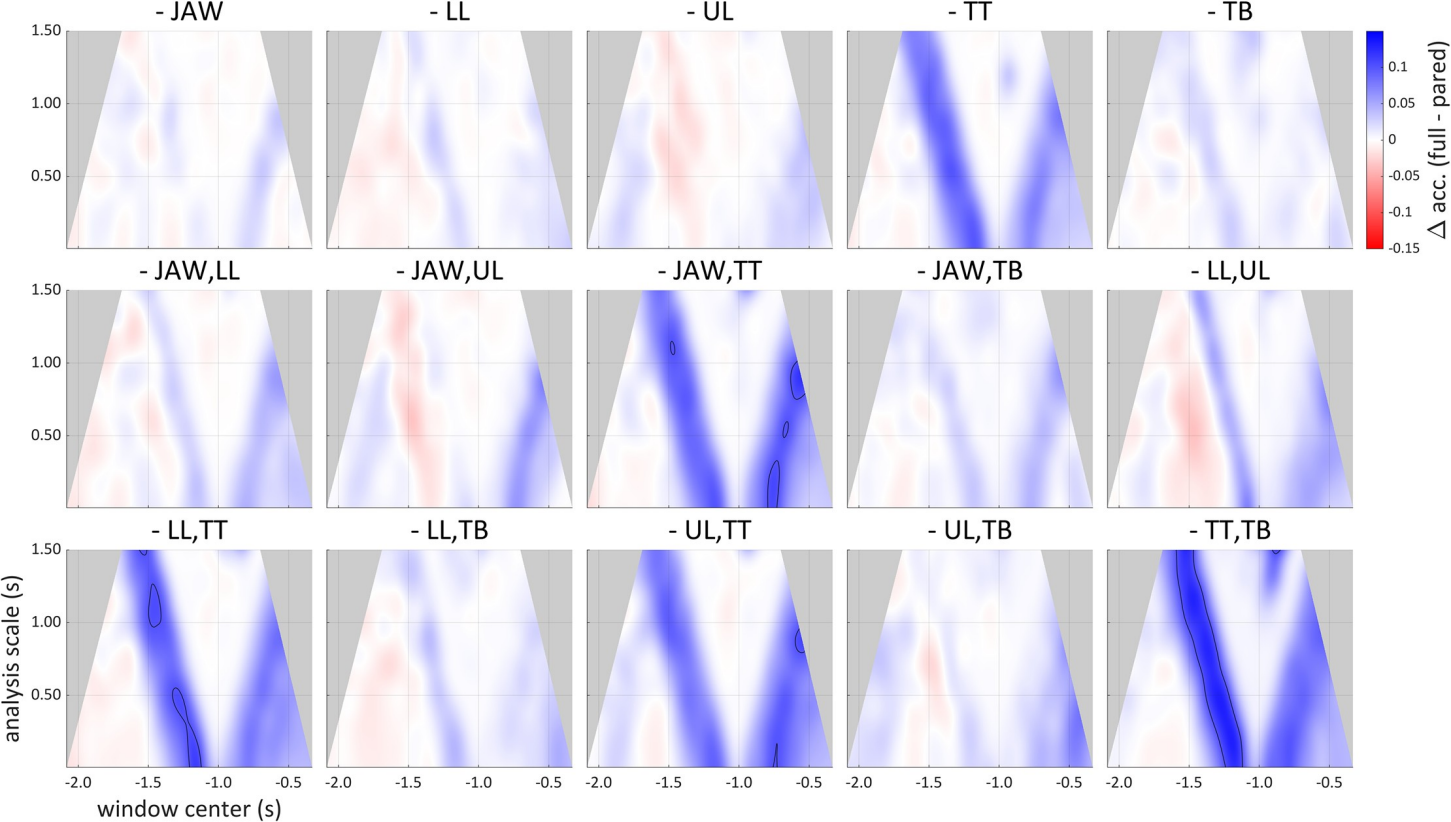
Differences of scalograms showing loss of classification accuracy obtained from paring subsets of signal dimensions. Accuracies from pared datasets are subtracted from the accuracy on the dataset with all articulatory signal dimensions included. Blue corresponds to a loss of accuracy. Contour lines indicate 10% accuracy differences. Top row: dimensions associated with a single articulator were pared. Middle, bottom rows: dimensions associated with two articulators were pared.

The scalographic analyses can be used to draw inferences regarding redundancy of signal dimensions. An example from the syllable production dataset is provided in Fig 11, which shows changes in accuracy associated with removal of the set of all channels related to a single articulator or pair of articulators, relative to the network accuracy obtained with all of the articulatory dimensions. The sets of dimensions removed singly or in pairs were those associated with the upper lip (UL), lower lip (LL), mandible (JAW), tongue tip (TT), or tongue body (TB). This analysis accomplishes a “spatial” localization of information, in the sense that signal dimensions constitute a high-dimensional space and particular subspaces correspond to physical parameters of the geometry of the vocal tract. Removing TT dimensions has the largest effect on accuracy, suggesting that the information in TT dimensions has the least redundancy with information in the other dimensions.

**Fig 12:**
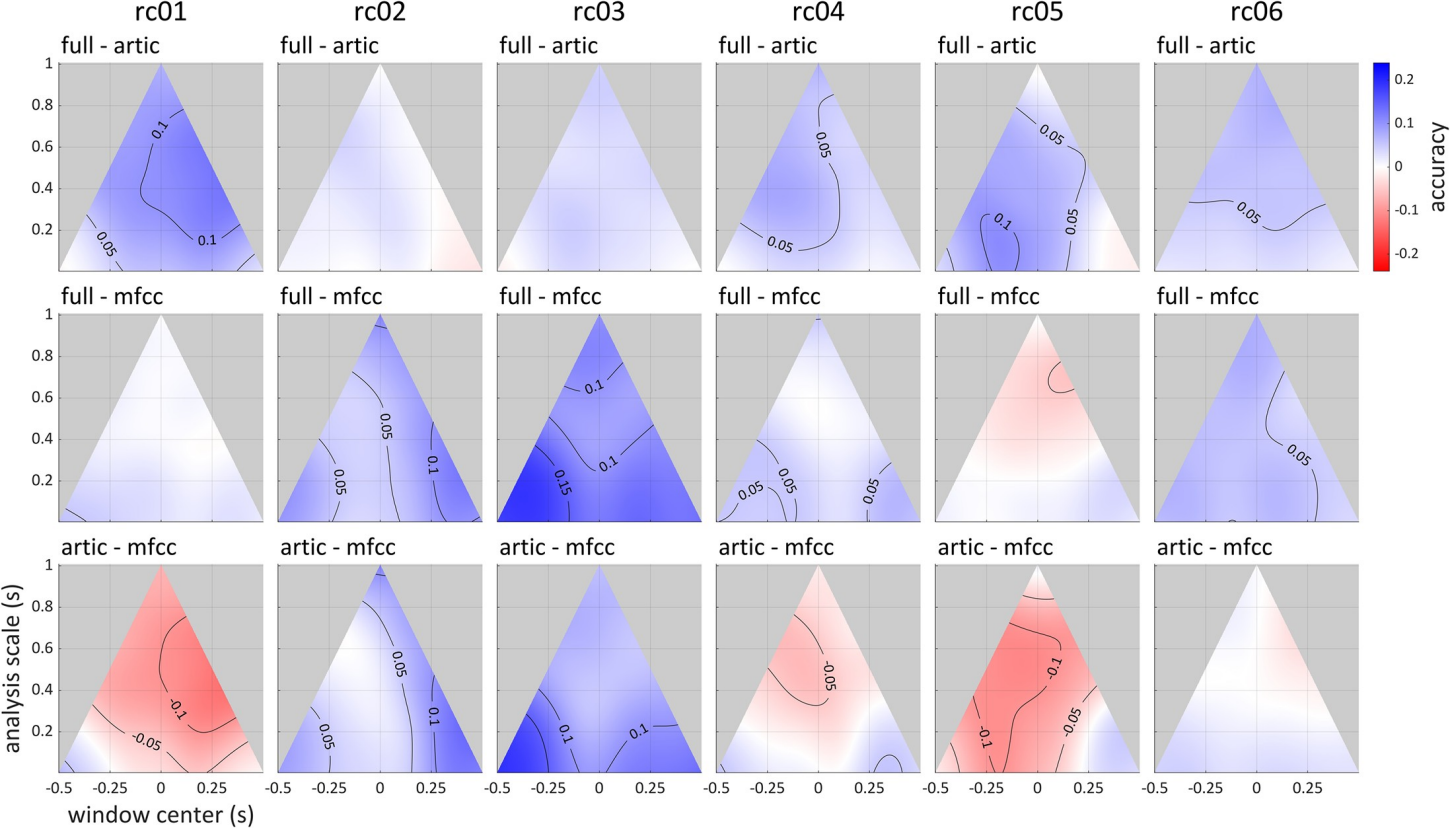
Differences of scalograms showing loss of classification accuracy obtained from removing acoustic (mfcc) or articulatory (artic) subsets of signal dimensions. Top row: articulatory dimensions removed; middle row: acoustic dimensions removed. Blue corresponds to a loss of accuracy. Bottom row: difference in accuracies between analyses restricted to articulatory or acoustic inputs.

In the relative clause dataset, signal dimension redundancy was investigated in relation to acoustic vs. articulatory subsets of signal dimensions. Fig 12 shows scalograms in which accuracies obtained with only articulatory or acoustic dimensions are subtracted from accuracies obtained with both dimensions. It also shows the difference between analyses restricted to articulatory or acoustic dimensions. There are two participants (rc01, rc05) who show a substantial accuracy reduction when articulatory dimensions were excluded but not when acoustic ones were; we may thus infer that, for these participants, the information in acoustic signals is mostly redundant with information in articulatory signals but not vice versa. Conversely, two other participants (rc02, rc03) exhibited substantial accuracy reduction with acoustic signal removal but not articulatory signal removal. A third pattern (rc06) shows similar accuracy reduction for both cases, suggesting either a greater degree of redundancy between acoustic and articulatory signals and/or complementarity between the two domains. These patterns are also evident when the difference of the articulatory and acoustic analyses are plotted, as in the bottom row of Fig 12.

## Methods

The following methods apply to both datasets, except where otherwise noted. The experimental designs used for collecting these data were described in section 1.3. The research was approved by the Cornell University Office of Research Integrity Assurance, under Protocol ID# 1106002289. Written consent was obtained from human subjects. Additional details not directly relevant to the current analyses are reported in [1] and [2] for the syllable and relative clause production tasks, respectively.

### Data processing and input

Articulatory data were collected at 400 Hz with an NDI Wave electromagnetic articulograph. Articulator sensors were located on the upper lip (UL), lower lip (LL), lower incisors (JAW), tongue tip 1cm from the apex (TT), and tongue body 5–6 cm from the apex (TB). Reference sensors on the nasion and mastoid processes were used for head movement correction. The articulator and reference sensors were lowpass filtered at 10 and 5 Hz, respectively (using 4^th^ order Butterworth filters).

Acoustic data were collected at 22050 Hz and were transformed to MFCC matrices using a 30 ms window and 5 ms time steps. A third-party MATLAB toolbox (Kamil Wojcicki, HTK MFCC MATLAB) was used to calculate MFCCs, with the following parameter values: frequency range: 300–5000 Hz; number of cepstral coefficients: 12; preemphasis factor: 0.97; number of filterbank channels: 20; cepstral sine lifter parameter: 22.

The maximal input signals to the networks and discriminant functions contained 24 dimensions of acoustic information (12 MFCC coefficients plus 12 ΔMFCC values) and 20 dimensions of articulator information, which were the horizontal and vertical positions of the articulator sensors, and the first differences of these. Thus there were 44 total input dimensions. Articulator positions and velocities were sampled at 5ms for network input and matched to MFCC window centers. All dimensions were z-score normalized by participant.

Signals in both datasets are aligned by participant to a reference point, which is defined as time 0. For the syllable production dataset, input signals were aligned to the initiation of the vocalic movement in the target form. This event was defined as the RMS velocity maximum in the tongue body sensor in the vicinity of the transition from pre-response vowel /i/ to the target vowel /a/. The beginning and ends of the input signals were determined as the earliest and latest times that were common to all trials. Movement initiations for onset /p/ and /t/ trials were estimated using standard procedures (see [1]), i.e. using 20% velocity thresholds applied to tongue tip vertical position for /t/ and lip aperture (Euclidean distance between UL and LL) for /p/.

For the relative clause dataset, input signals were aligned to the ends of the relevant acoustic intervals demarcating the start and end of the relative clause. For example, consider the sentence: *A Mr*. *Hobb*, [_pre_ *who knows Mr*. *Rodd*] _post_, *often plays tennis*. In the case of the pre-clause boundary, the alignment point is the end of the acoustic interval associated with *Mr*. *Hobb*; in the case of the post-clause boundary, the alignment point is the end of *Mr*. *Rodd*. The acoustic intervals were determined by forced alignment with Kaldi [47], using the procedures described in [2].

### Machine learning algorithm parameters

The discriminant analyses used a very simple linear discriminant function which assumes a Gaussian mixture distribution in the input data, with the same covariance matrix for each class. The model is trained to minimize the expected classification cost over categories, where each cost is the product of the posterior probability of the category given the data and the classification cost (0 for correct classification, 1 for incorrect classification). Prior probabilities of categories were uniform, and a regularization of γ = 0.5 was used. In an preliminary version of this manuscript we reported discriminant analyses with no regularization and observed a large decrease in accuracy at small scales (from 0.050 to 0.100 s). We suspected that this was due to characteristics of the covariance matrix in such windows, and a reviewer correctly suggested that mild regularization would solve the issue.

The hyperparameter spaces that define the neural network and its training procedures are too large to investigate systematically, and we examine issues regarding hyperparameter analysis further in the section General Discussion. Decisions regarding network architecture and training parameters were made on the basis of experience from previous work [1] and additional exploratory testing of a small set of parameters on a subset of data. The classification networks used here consisted of an input layer followed by two layers of 350 bidirectional LSTM units. Each biLSTM layer was followed by a 50% dropout layer. The last dropout layer was followed by a fully connected layer, a SoftMax layer, and a classification layer with a cross-entropy loss function.

The network training procedure used an adaptive moment estimation optimizer (Adam) with the following parameters: gradient threshold: 1.0; initial learning rate: 0.0001; L2 regularization 0.01. Optimization was limited to a maximum of 1000 epochs, and mini-batch sizes of 50 were used (an iteration is one step of the optimization algorithm, including weight updates, applied to a mini-batch, and an epoch is a full pass of the training algorithm over the training set). The order of sequences and their assignment to mini-batches were shuffled every epoch. The validation frequency and patience were 5 and 50 iterations, which means that validation loss is calculated every 5 iterations and training stops if the validation loss does not decrease over 50 iterations.

### Multi-scale analysis procedures and visualization

Network and discriminant analyses are conducted separately for each participant. For the syllable production dataset, analysis windows were centered at 50 ms steps from the beginning of the signal. For the relative clause dataset, analysis windows were centered at the alignment point and taken in 50 ms steps up to 500 ms before and after the alignment point. The ±500 ms restriction in the relative clause dataset was imposed due to greater variation in speech rate in that dataset, which results in greater misalignment further from the alignment point. Window sizes were chosen by making a list of all window sizes with an odd number of frames, from 1 frame up to the maximal number, which is determined by the period of time over which signals are available. Window candidates were restricted to windows with odd numbers of frames so that signals can be centered exactly on a given frame. The formula *i*_sel_ = ceil[*e^b(i−N)^*] was used to select indices of the list, where *i* is the index of the list of odd frames, *N* is the number of elements of the list, and *b* is a parameter which controls the exponential growth of the list indices. A value of 0.1 was used for *b*, but other values may be useful in different contexts. The ceiling function was applied to the output and all unique indices were selected. The purpose of using an exponential function to select window sizes is to provide more closely spaced (approximately linearly spaced) window sizes at small scales, while using larger spacing for larger scales. No windows are analyzed which would extend beyond the available time horizon of the signals and hence would require zero-padding. We avoid this because in pilot work we observed that zero-padding negatively affected network performance.

For each analysis window, multiple repetitions of the partitioning and training procedures (steps 2–4 of Fig 2) were conducted. For the scalographic analyses reported the results section, five repetitions were used, except for the subtractive analyses of the syllable production dataset, where two repetitions were used. For each repetition, the data were randomly partitioned into training and test sets. The numbers of each category were exactly balanced in all training and test sets, with one exception: an oversight in conducting analyses of the syllable production dataset sometimes led to a difference of one between category counts in the test set. We do not believe this is problematic because it always constitutes a less than 1% deviation from exact balance. For the relative clause production dataset, the target category (relative clause type) was exactly balanced within training and test sets, and the identity of the pre-boundary coda consonant (/b/ or /d/, cf. *Mr*. *Hobb* vs. *Mr*. *Hodd*) was balanced within target categories.

In order to keep the training and test datasets as large as possible, the test data were also used for validation during training; thus the test data can determine when the training stops, and may bias the accuracy on classification of the test data. To check that the identity of test and validation sets did not result in a large bias, we conducted analyses on a subset of analysis windows for participant sy01 in the syllable production dataset, using a 1/3 split of disjoint training, validation, and test datasets for each of five repetitions. A paired comparison of average accuracies found a small difference in accuracy of 0.011 (95% confidence interval: [0.0037, 0.0182]). Hence the use of test data for stopping training may indeed create a bias, but this bias is relatively small. Future studies which aim to precisely localize temporal distributions of information should use disjoint test and validation sets.

To visualize the results of the multi-scale analyses, we plot scalograms, which are pseudocolor plots of matrices of average accuracies. A raw scalogram (i.e. not smoothed, not interpolated) was shown in Fig 2, step 6. Note that because our sampling steps in the window scale dimension are exponentially spaced, the vertical sizes of pixels in the raw scalogram increase with scale. In order to smooth the raw scalograms, a 2D-Gaussian filter is applied, with standard deviations of 100 ms in both the window center and window scale dimensions. These standard deviation parameters are arbitrary and can be adjusted independently to control the degree of smoothing in both center and scale dimensions. In order to obtain more finely sampled representations of accuracy, cubic spline interpolation was applied to the smoothed scalogram, using a grid of 1 ms steps in the window center dimension and 5 ms steps in the window scale dimension. In all cases only points in the scalogram are retained which are within a polygonal region defined by the original analysis window parameters.

### Validation with simulated data

**Fig 13:**
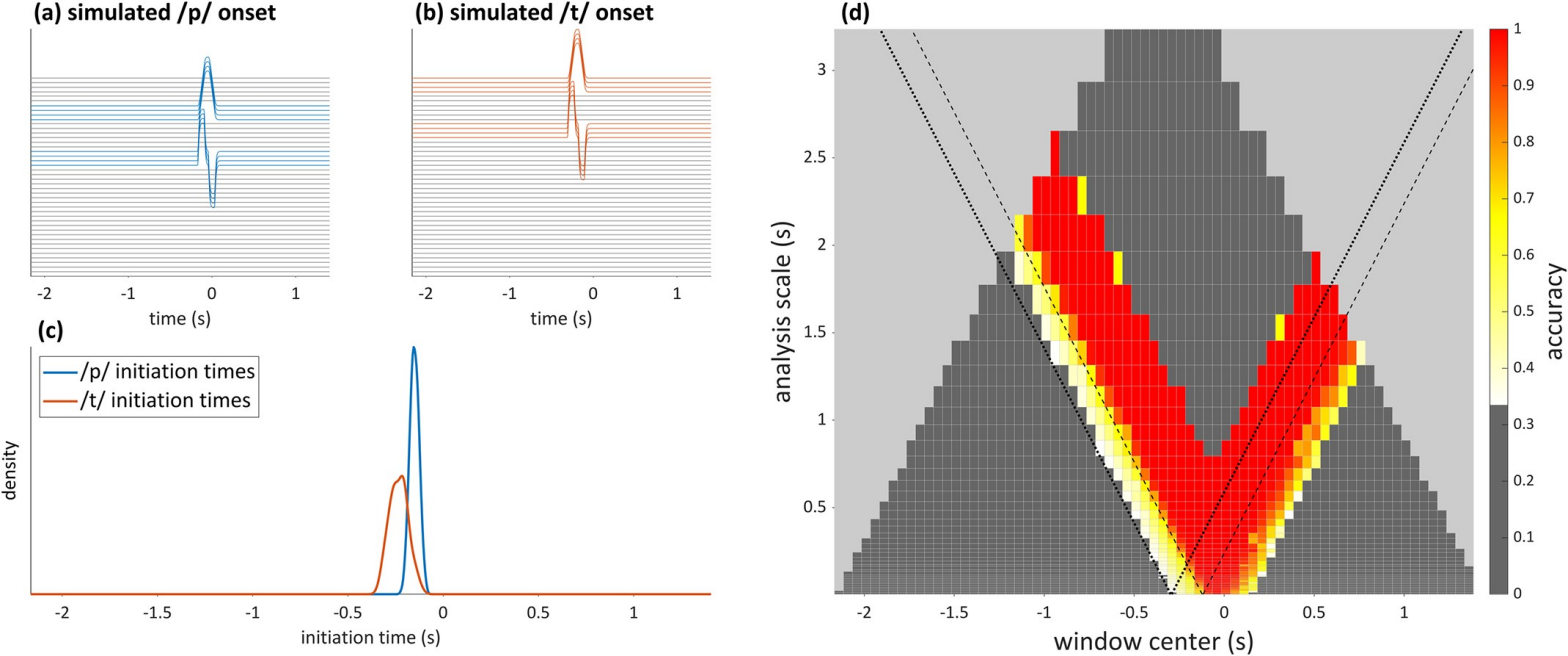
Illustration of decline in network accuracy associated with large window sizes, using simulated data. (a, b) Simulated articulatory signals associated with simulated /p/ and /t/ onsets. (c) Gaussian kernel smoothed density distributions of movement initiation times matching empirical distributions. (d) Scalogram showing accuracy decrease at large analysis scales.

An important point to emphasize is that network architecture and training parameters are held constant across analyses, and thus are not expected to perform equally well for all scales. Indeed, a substantial degradation of performance for large analysis windows was observed in the syllable production dataset. To test whether this degradation was caused by some unknown aspects of the data, we conducted the analysis procedures on a simulated dataset which mimicked the statistical properties of movement onset times in the syllable production dataset. In this simulated dataset, we constructed input signals such that the categories /p/ and /t/ were associated with 200 ms intervals in which signal values were ramped up from 0 to 1 and back down to 0, in the horizontal and vertical UL/LL and TT/TB channels, respectively. The first differences of these were included as well. The ramping was controlled by a driven, critically damped harmonic oscillator, which accords with a popular model of articulator kinematics [40]. All other dimensions were set to zero. An example is shown in Fig 13. The network training procedures were applied to simulated data, and scalograms were constructed using the procedures described above. A reduction in accuracy for large analysis windows was observed, similar to what is seen with empirical data. The results on simulated data indicate that the low accuracy for large windows is a consequence of the network architecture or training parameters, rather than being due to some unknown characteristic of the data. It may be possible to improve the network performance for large windows by using scale-dependent network architecture/training parameters.

**Fig 14:**
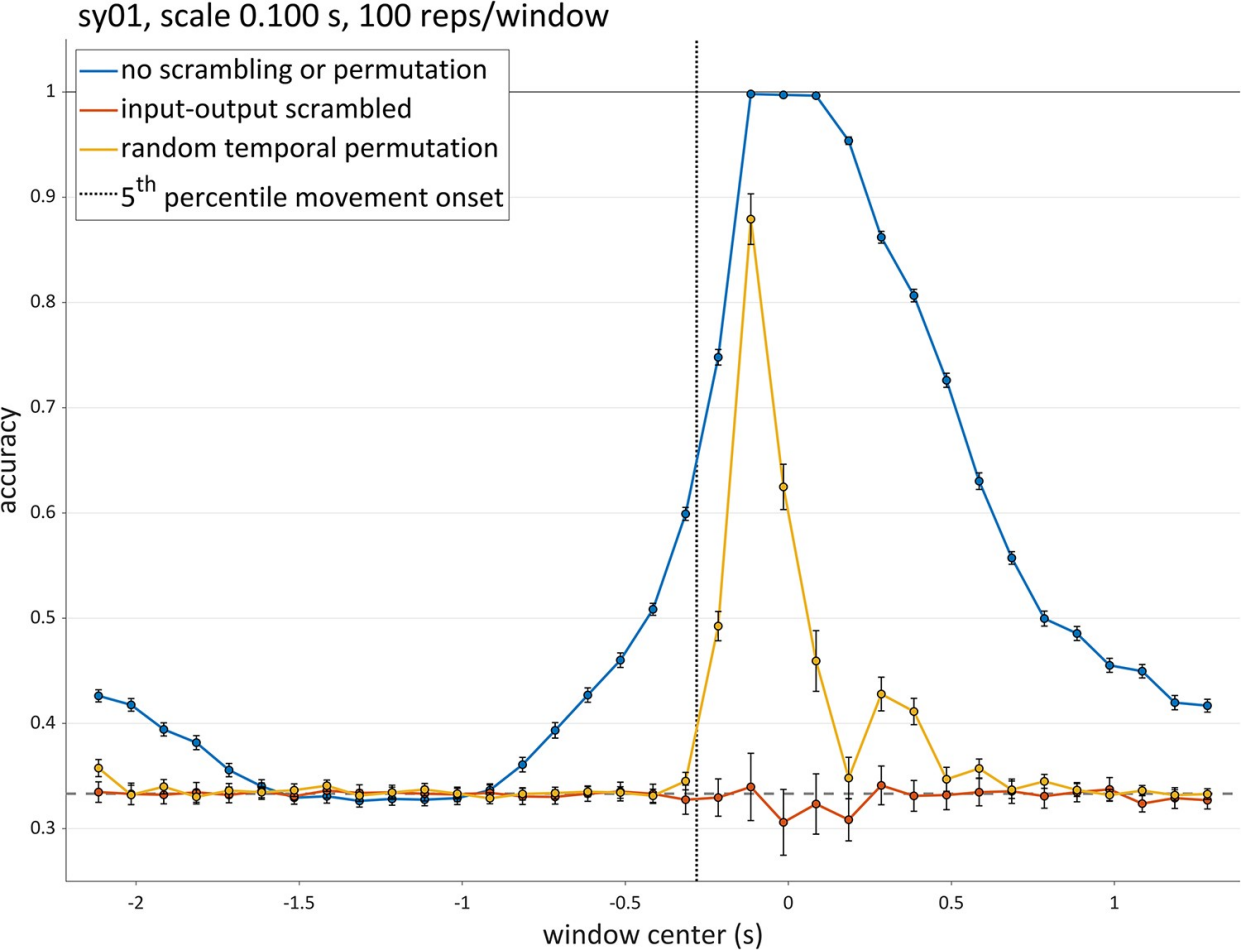
Accuracy confidence intervals for one speaker and scale (100 ms). Blue dots: analyses conducted on original data; orange dots: input-output mappings scrambled in training set; yellow dots: frames in each analysis window of training set randomly permuted. Error bars show 99% confidence intervals. Horizontal dashed line represents chance accuracy. Vertical dotted line shows the 5% percentile of movement initiation.

To assess whether accuracies for a given analysis window are reliably above chance, a simple and straightforward way to obtain a confidence interval is to calculate the standard error of the accuracy for each window. Fig 14 shows mean accuracy (blue dots) and 99% confidence intervals (± 2.57 s.e.) for analyses of 100 ms windows for speaker sy01 of the syllable production dataset. In these analyses, 100 training/test repetitions were conducted for each window. The confidence intervals are quite narrow, indicating that with this many repetitions, the accuracy can be estimated fairly precisely. It should be noted, however, that this approach assumes a normally distributed accuracy and is not entirely appropriate when accuracy is close to its ceiling (100%) or in the vicinity of chance. If more exact determination of confidence is desired, confidence intervals of parameters from a logistic regression could be obtained instead.

In order to verify that accuracy does indeed indicate the presence of category-related information, we conducted analyses in which the mappings from inputs to output categories were randomly scrambled in the training data. We hypothesized that this scrambling would fully prevent the networks from learning generalizable input-output mappings and thus would result in chance performance for all windows. As shown in Fig 14 (orange dots), this hypothesis was partly confirmed: no analysis windows exhibited accuracies on test data which were reliably above chance. However, there is an epoch of marginally *below* chance performance in the vicinity of the alignment point. This could be due to the fact that with scrambled input-output mappings the network learns incorrect mappings, and subsequently is biased to make incorrect decisions on the test data.

In order to test whether the temporal order of input frames is important for the network to learn generalizable input-output mappings, we conducted analyses in which the frames in each analysis window were randomly permuted in the training data. We hypothesized that this would diminish network accuracy relative to the non-permuted data, but still would result in above-chance levels of accuracy. The motivation for this prediction is that the same spatial information regarding the articulatory and acoustic signal dimensions is present in the permuted data, despite the fact that the order in which the network receives that information is random. As can be seen in Fig 14 (yellow dots), the hypothesized impact was indeed observed during the epoch of time from about 250 ms before to 500 ms after the alignment point. In this epoch, the target syllable is being produced. However, the temporal scrambling resulted in chance accuracy in earlier and later windows, where the analyses of non-scrambled, non-permuted data obtained substantially above chance accuracy in the original analyses. This unexpected result may have the following interpretation: the category-related information which is present during the pre-response vowel and after the target response may be predominantly or essentially temporal in nature. In other words, it is not merely information regarding the states of the vocal tract but rather information regarding the temporal evolution of that state which the network learns to use to classify the onset consonant categories. This suggests that the multi-scale analysis procedure we have developed might provide a way to dissociate the contributions of temporal information from spatial information in relation to hypothesized categories.

## General discussion

The results above show that the accuracy scalogram can allow for a variety of theoretically-relevant inferences to be drawn regarding how linguistic categories are manifested in speech signals. These inferences include aspects of signals that relate to the temporal distribution of information, asymmetries between information located before vs. after particular events, the potential scale-dependence of relevant information, and differences in category-related information that involve the sources of signals themselves (e.g. acoustic vs. articulatory information, or different articulators). In particular, it was observed in the syllable dataset that there is information which can be used to identify onset consonant categories both well before movement initiation and also after the production of a syllable. In this case, the multi-scale nature of the analysis showed that this information is available even on very small scales (see Fig 7, left), and that increasing analysis scale does not provide additional improvement in classification accuracy. In contrast, in the analysis of the relative clause data, a fairly strong scale-dependence of accuracy was observed: as analysis windows extended further from the clause boundary, accuracy increased (see **Fig 9**). This suggests that additional information relevant to distinguishing clause types is available at times that are distant from the boundary. These sorts of inferences depend crucially on conducting analyses across a range of scales, and it stands to reason that an analysis which arbitrarily examined only a single scale would fail to identify such patterns.

The analysis method is also well-suited to investigating how sources of information in a signal may differ or interact in the extent to which they provide category-related information. For example, in the analyses of the syllable production data in which individual articulators and pairs of articulators were removed, it was observed that removal of tongue tip (TT)-related channels resulted in the greatest accuracy loss (see **Fig 11**).

Another noteworthy aspect of our method is that it in some cases it revealed differences between speakers. For example, in the analysis of accuracy loss in the relative clause dataset when either articulatory or acoustic dimensions were removed from the full input (see **Fig 12**), it was observed that the effects of paring acoustic/articulatory dimensions is speaker-dependent. One possible explanation for this is that variability and/or stationarity in these subsets of dimensions may differ between speakers; indeed, speaker-specific differences in acoustic vs. articulatory variability in production of vowels have been reported in [48]. It is possible that these differences in variability/stationary have consequences for accuracy that are independent of mutual dependency, and this constitutes another caveat in interpretation of results.

There are a number of points that are important to keep in mind when interpreting accuracy scalograms. First, machine learning algorithms cannot obtain reliably above-chance accuracy on test data when no category-related information exists in training inputs. In other words, the results are not due to overfitting. The qualifier *reliably* is important because random variation in partitioning and training procedures results in statistical fluctuations in classification accuracy.

Second, the algorithms are in general expected to obtain greater classification accuracy when more category-related information is present, up to the limit of maximal accuracy. However, this does not entail that there is a simple functional relation between classification accuracy and the amount of mutual information between categories and signals. In fact, we saw that there are circumstances in which the assumption that more information results in higher accuracy is clearly invalid; these circumstances may arise when the learning algorithm is not well-suited for the classification task.

Third, drawing inferences about category-related information from classification accuracy is only sensible when the hypothesized categories are approximately balanced within both training and test sets. We focus on classification accuracy because of its interpretability, but it is worth pointing out that the neural network classifiers are trained by backpropagation of cross-entropy loss. In cases where there is a substantial imbalance in the numbers of categories in the data, cross-entropy may be preferable for drawing inferences. In all cases, the training and test data should have similar distributions of categories, whether those distributions are balanced or not.

Fourth, inferences regarding the temporal extent of category-related information should be made relative to an alignment point in the signals. These inferences necessarily depend on domain-specific assumptions regarding the processes which are responsible for generating the signals, as well as limitations of the machine learning algorithm that is used. For both of the datasets we examined, and likely in general for speech data, greater temporal distance from the alignment point is associated with greater temporal misalignment of the signals and potentially worse classification accuracy. The biLSTM neural network architecture is more robust to this misalignment, while the LDA analyses are more adversely affected by it.

Fifth, the analyses can at most provide lower bounds on the temporal extent of category-related information. No learning algorithm can be proven to be optimal for data of the sort we examine, i.e. nonstationary speech data with unknown generating processes. Moreover, the hyperparameter space of the neural network algorithm we use is very large and we cannot know if the particular parameters we employ are optimal or even close to optimal. Thus classification accuracies obtained from this algorithm are almost certainly under-estimates of the presence of category-related information. In other words, there is always a possibility that there exists a better algorithm. Hence above-chance accuracy in the scalogram should be interpreted to provide minimal ranges of the temporal distribution of category-related information in signals: for a given analysis scale, the range of analysis window centers over which above-chance accuracy is reliably obtained indicates that category-related information is present; however, such information may still be present earlier or later and yet not detected due to sub-optimality of the classifier. Furthermore, our neural network hyperparameters cannot be assumed to be equally suboptimal across scales, and hence some of the variation we observe across analysis scales may be due to differences in how well-suited the hyperparameters are for a given scale. Below we elaborate further on the potential benefits and challenges that arise in optimization of hyperparameters. Finally, inferences about the temporal extent of category-related information from scalograms should be interpreted in relation to a set of observations, rather than in relation to specific categories. Classification accuracy derives from the ability of a classifier to learn to distinguish members of set of categories associated with an ensemble of observations. This is different from identifying information specific to a particular category in a single observation. The methods can in theory be adapted to this task—for example by examining the posterior probabilities of the discriminant analysis classification model predictions for each observation, or alternatively the softmax category outputs of the neural network—but we do not pursue this application here.

It is important to note that we see no reason to impose restrictions on the particular machine learning algorithms that might be employed in a scalographic context. Both our neural network and discriminant analysis approaches are examples of supervised learning, in which the algorithm is provided information regarding the "correct" (or hypothesized) categories that are associated with input signals. We used supervised learning techniques because we believe these can be more directly applied to a specific set of hypothesized categories than would be the case for alternatives which use unsupervised learning or reinforcement learning. For example, in an unsupervised learning context, where the algorithm might select some number of categories by clustering signals, there is no guarantee that the hypothesized categories of interest are the ones which the algorithm will be most inclined to learn. Indeed, other sets of categories which may manifest more strongly in the input could easily dominate the outcome. Thus the supervised approach allows us to focus on just one dimension of categorical information and ignore others. It would nonetheless be interesting to see how the categories which are learned in unsupervised approaches vary as a function of analysis window location and scale, and whether these would in fact correspond to hypothesized categories of interest.

A desirable next step in the pursuit of neural network-based multiscale analysis is to examine how the optimal network architecture and training hyperparameters vary as a function of window scale. Knowledge of this variation is desirable because it would further inform the interpretation of scale-related changes in accuracy. Indeed, to an unknown extent, the effects of analysis scale that we observe might be attributed to differences in the extent to which the parameters we use are optimal for a given scale. This suggests that it would be useful to characterize how accuracy is reduced for each scale, compared to the accuracy that might be obtained with hyperparameters that are optimized for that scale. One way to accomplish this would be express accuracy for each center/scale, relative to the maximum accuracy that was obtained at a particular center.

However, there are numerous challenges to obtaining a scale-specific hyperparameter optimization of this sort. First, since there are at least eight parameters of the neural network architecture and training algorithm, six of which are continuous (see Methods section), a brute force search of even a very coarse three-point grid in each hyper parameter dimension would require training many networks. Indeed, three points would be not be sufficient for this search, particularly in dimensions in which plausible parameter values span orders of magnitude, such as learning rates or L2 regularization. Second, if we did conduct a brute force search, it might be quite difficult to interpret the results, since it is likely that many combinations of parameters will result in effectively the same average accuracy—in effect the optimal parameters occupy a hypersurface that will be difficult to examine. Third, if we were to restrict the hyperparameter analysis to a subspace of parameters and sample with finer resolution, we might draw misleading inferences by ignoring interactions with non-varied parameters. Any statement regarding the optimal parameters in a given subspace must be qualified as conditional on specific values of non-varied parameters. Fourth, even if we did conduct an analysis of the scale dependence of the optimal hyperparameters on a particular dataset from one speaker, it is unclear whether it would generalize to other datasets; such analyses would need to be conducted on all datasets, which is logistically impractical for us. Perhaps some of the logistical issues could be addressed by optimizing hyperparameters with simulated data, as we did in the section "Validation with simulated data". This method could be informative but it requires assumptions which are not tenable regarding equivalence between the simulated data and empirical data.

## Conclusion

The multiscale analysis technique presented here was shown to be useful for localizing category-related information in articulatory and acoustic signals. The approach allows for lower limits to be inferred regarding the temporal range of category-related information, and it can be used to localize information to specific signal dimensions or subsets of dimensions. However, it is important to emphasize that no analytical relation is derived between network accuracy and mutual information between signals and categories, which is ultimately the quantity of interest; moreover, the analysis is necessarily sub-optimal due to the high dimensionality of the parameter space for network design and training. Future work may benefit from additional exploration of hyperparameters or alternative signal dimensions. For example, work in progress indicates that the autocorrelogram can be used to provide input that relates to characteristics of vocal fold vibration. Moreover, other network architectures or machine-learning algorithms might be usefully substituted for the biLSTM layers used here. Nonetheless, scalographic analysis can be generally used to address theoretical questions using empirical data, and can do so without requiring a priori assumptions about the mechanisms which relate signals and categories.
